# Cross-Sectional Relationships Between Gender, Disordered Eating Behaviors, and Suicide Among High Schoolers in Colorado

**DOI:** 10.3390/ijerph22020152

**Published:** 2025-01-24

**Authors:** Avery M. Anderson, Sophie Rosenberg, Heather E. Schier, Sarah K. Eskew, Scott B. Harpin, Ashley Brooks-Russell, Christina J. Sun

**Affiliations:** 1 College of Nursing, University of Colorado Anschutz Medical Campus, Aurora, CO 80045, USA; 2 Department of Community & Behavioral Health, Colorado School of Public Health, University of Colorado Anschutz Medical Campus, Aurora, CO 80045, USAashley.brooks-russell@cuanschutz.edu (A.B.-R.); 3 Nutrition and Foods Program, Appalachian State University, Boone, NC 28608, USA

**Keywords:** suicide, disordered eating, gender identity, gender dysphoria, adolescent mental health, transgender, health psychology

## Abstract

Though transgender and gender diverse (TGD) youth are disproportionately affected by suicide and disordered eating, little research has explored the relationship between the two using state-level data. This exploratory study examined whether disordered eating behaviors moderate the observed relationship between gender and suicide among adolescents. Multivariate logistic regression was performed on the population-based 2023 Healthy Kids Colorado Survey (HKCS) data (*N* = 49,989) to test whether the odds of suicide ideation and attempt differed by gender groups, and modeling was carried out to examine the moderation of these relationships by disordered eating behaviors. Additionally, analyses were replicated among only gender minority groups (*n* = 2486). Compared to cismale students, the prevalence of disordered eating was higher among all other gender identities. There was a significantly higher risk of suicidal ideation and attempts among transfemale, transmale, nonbinary and gender-questioning students. Disordered eating did not significantly moderate the relationship between gender and suicide outcomes. These findings underscore the heightened vulnerability of TGD youth to disordered eating and suicidal behaviors while suggesting that disordered eating may not be a pathway through which gender relates to suicide outcomes.

## 1. Introduction

Suicide and disordered eating are two health issues that disproportionately affect transgender and gender diverse (TGD) adolescents. Existing research consistently finds that TGD youth have a greater suicide risk compared with cisgender youth, including cisgender lesbian, gay, bisexual, queer, or gender-questioning youth [1], and many representative population-based surveys of youth in the United States (US) still do not include appropriate measurement of gender identity. Thus, it remains challenging to assess suicide rates in this subpopulation of youth, compare rates to cisgender peers, and explore factors that affect the relationship between gender identity and suicide outcomes.

Disordered eating behaviors are defined by a broad range of eating-related problems (e.g., binge eating, weight control vomiting, food restriction, dieting, emotional eating, etc.), which are less frequent or of less severity than the Diagnostic and Statistical Manual of Mental Disorders Fifth Edition (DSM-5) diagnostic criteria for eating disorders [2,3]. Similarly to suicide risk, the prevalence of disordered eating behaviors is disproportionally elevated among TGD youth, with 48% of adolescents aged 14–18 reporting fasting within the past year, 42% reporting binge eating, 18% reporting purging, 7% reporting the use of diet pills, and 5% reporting the use of laxatives [4]. Since disordered eating behaviors are often thought to be employed as mechanisms for coping and harnessing control, emerging research suggests food restriction and/or compensatory eating are means of coping with gender-related distress among TGD adolescents [5].

The intersection of disordered eating and gender dysphoria presents unique and alarming challenges [6]. A subset of TGD adolescents may experience gender dysphoria, characterized by a profound discomfort with one’s assigned gender at birth, which can lead to a pervasive sense of loss of control over one’s body and identity [2,7]. To cope with this sense of a lack of control, restrictive disordered eating behaviors can be viewed as one thing they can control [8]. However, there is also evidence that loss of control/binge eating is more common among TGD individuals than their cisgender counterparts [9,10]. Disordered eating as an act alone may be used to “punish” oneself. In some cases, it may also function as a method of manipulating body weight and body composition distribution or disrupting reproductive hormones (e.g., binge eating to “bulk”; restrictive eating to minimize “feminine” curves or disrupt menstruation) [5,11]. To some degree, disordered eating patterns may facilitate gender affirmation, initiating a vicious positive feedback loop [12,13]. While the relationships between gender dysphoria and disordered eating are complex, there are also factors that may contribute to the development of disordered eating in TGD adolescents that are independent of experiencing gender dysphoria [14].

Inconsistent findings have emerged from comparing TGD individuals who access gender-affirming care (e.g., hormone therapy) with those who do not and the prevalence of subsequent disordered eating behaviors [15,16]. While disordered eating in TGD populations may be conceptualized as a maladaptive coping strategy for managing gender dysphoria, it is also likely a maladaptive coping strategy adopted in response to other forms of gender-related distress, such as stigma, discrimination, abuse, and internalized transphobia [6,17,18,19]. The literature describes significant associations between these minority stressors and increased suicide outcomes among TGD adolescents [20].

To better understand the relationship between gender and suicide through intervenable factors, the purpose of this exploratory study is to examine whether disordered eating behavior moderates the observed relationship between gender and suicide among Colorado public high school students. A more accurate assessment of eating behaviors may be an avenue through which disparate suicide outcomes by gender can be addressed. The aims of this study are to (1) compare disordered eating behavior and suicide outcomes by gender and (2) examine disordered eating behavior as a moderator of the relationships between gender and suicide outcomes.

## 2. Materials and Methods

Data were collected from the 2023 Healthy Kids Colorado Survey (HKCS), a biennial cross-sectional population-based survey of Colorado public high school students. The survey was self-administered online in classrooms during a regular class period. Teachers oversaw the survey administration and were directed to read a script and show a short video providing instructions for the students. Participation was voluntary and approved by parents; no identifying information was collected from the students. This study was approved by the Colorado Multiple Institutional Review Board. The HKCS instrument includes several questions from the Youth Risk Behavior Surveillance System (YRBS) [21], with the 2023 HKCS being the first to include the disordered eating behavior question.

Demographic variables included students’ grade level in school (9th, 10th, 11th, or 12th) and race/ethnicity (categorized as non-Hispanic White, non-Hispanic Black/African American, Hispanic, multi-racial (two or more responses), and all others (American Indian/Alaska Native, Asian American, Native Hawaiian/Pacific Islander, Middle Eastern/North African, and other identities not listed). Students also reported the highest level of their mother’s education (categorized as high school or less, some college, and college graduate or more).

Gender identity was classified into six categories based on their responses to two questions: “What is your gender identity?” and “Some people describe themselves as transgender when their sex at birth does not match the way they think or feel about their gender. Are you transgender?” Students who responded “no, I am not transgender” and “female” or “male” were categorized as cisfemale or cismale, respectively. Those who responded “Yes, I am transgender” and “Female” or “Male” were categorized as transfemale or transmale, respectively. If students responded that their gender identity was nonbinary, they were categorized as nonbinary, while students who reported that they are not sure about their gender or identify as a gender not listed were classified as questioning.

Disordered eating was assessed using the YRBS question “During the past 30 days, did you try to lose weight or keep from gaining weight by going without eating for 24 h or more; taking any diet pills, powders, or liquids; vomiting or taking laxatives; or skipping meals?” The response options were yes/no.

Suicide outcomes were assessed using the questions “During the past 12 months, did you ever seriously consider attempting suicide?” (suicidal ideation; yes/no response) and “During the past 12 months, how many times did you actually attempt suicide?” (suicide attempt). Response options for suicide attempt were dichotomized to 0 and greater than or equal to 1.

All analyses were performed using SAS 9.4 (SAS Institute, Inc., Cary, NC). Descriptive statistics for suicidal ideation and attempt were compared according to demographic characteristics and disordered eating. Pearson’s chi-squared test was used to make determinations about differences in demographic groups. Multivariate logistic regression was performed to test the odds of suicidal ideation and attempt by all gender groups. Then, modeling examined the moderation of these relationships by disordered eating behaviors. Follow-up multivariate logistic regression was performed to compare the odds of suicidal ideation and attempt among only the gender minority groups and, likewise, to test moderation effects. Covariates for models 1–4 included grade, race/ethnicity, and mother’s education. The estimated odds ratio and 95% confidence intervals were reported. Confidence intervals that do not include 1.0 are statistically significant at *p* < 0.05.

## 3. Results

In the sample (*N* = 49,989), 48.3% were cismale, 46.0% were cisfemale, 1.8% were nonbinary, 1.0% were transmale, 0.4% were transfemale, and 2.5% were questioning (Table 1). Of the sample, 21.9% reported disordered eating behavior. Just over 1 in 10 (11.1%) reported suicidal ideation, and 1 in 20 (5.5%) had attempted suicide in the past 12 months.

**Figure 1:**
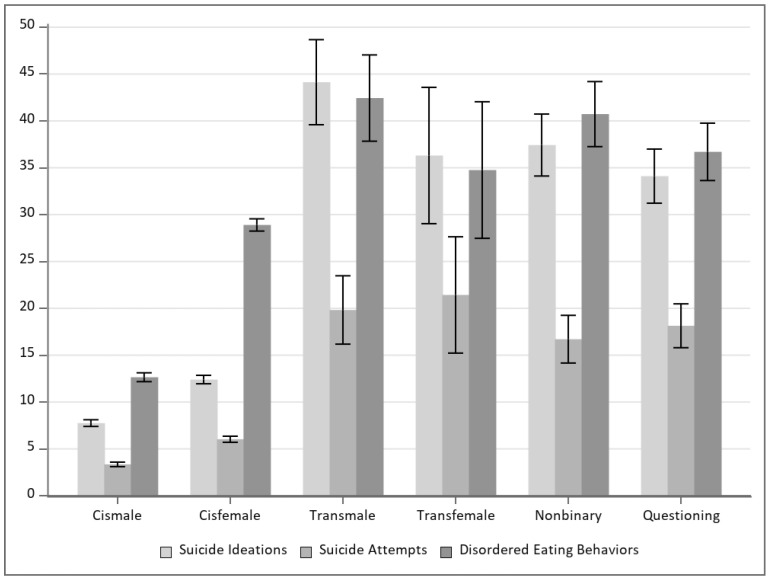
Prevalence of disordered eating behaviors and suicide outcomes by gender identity.

There was a significantly higher prevalence of disordered eating behavior, suicidal ideation, and suicide attempt particularly among transfemale, transmale, nonbinary, and questioning students (Figure 1). Disordered eating was also associated with suicide risk, as 15.3% of those with disordered eating reported having attempted suicide, as compared to 2.8% of those without disordered eating.

In model 1, the main effects of gender on suicidal ideation and suicide attempt were significant, such that, compared to cismale students, the odds of both suicide outcomes were significantly higher among all other gender identities (Table 2). For example, the odds of a student having suicide ideation are 9.6 times more likely in transmale students compared to their cismale counterparts after adjusting for grade, race, and mothers’ education. Likewise, the odds of a student attempting suicide are 7.1 times more likely in transmale students compared to their cismale counterparts after adjusting for grade, race, and mothers’ education. The odds ratios (Table 2, model 1) demonstrate significantly elevated risk among TGD adolescents compared to their cisgender peers. Disordered eating was added to model 2, as well as the interaction between gender and disordered eating. Disordered eating behaviors significantly moderate the relationship between gender and suicidal ideation and suicide attempt for cisfemales (suicidal ideation adjusted odds ratio [aOR]: 1.6, 95% confidence interval [CI]: 1.3–1.9; suicide attempt aOR: 1.5, 95%, CI: 1.2–1.9) but not for other genders.

In the gender minority subsample models (n = 2486; Table 2), transfemale, transmales, and nonbinary students were compared to gender-questioning students (excluding cismales and cisfemales). In the initial model (model 3), transmale students were significantly more likely to report suicidal ideation, but not suicide attempt, compared to gender-questioning students. It was found that the odds of a student having suicide ideation is 1.5 times more likely in transmale students compared to gender-questioning students after adjusting for grade, race, and mothers’ education. However, this relationship was attenuated by the inclusion of disordered eating (model 4), which was significantly related to suicidal ideation (aOR 3.1, 95% CI: 2.3–4.3). In the model for suicide attempt, a similar finding emerged, with significant main effects for disordered eating behaviors on suicide attempts (aOR 3.7, 95% CI: 2.4–5.5) and a lack of significant main effects for gender or the interaction between gender and disordered eating. Thus, moderation effects for disordered eating on the relationship between gender and suicide outcomes were not observed in this gender minority subsample.

## 4. Discussion

This study contributes a nuanced understanding of the complex interplay between gender identity, disordered eating behaviors, and suicide risk among high school students in Colorado. Uniquely, this sample size (*N* = 49,989) allowed for comparisons between cisgender and TGD groups as well as specific gender identities under the umbrella term of TGD. The findings underscore the heightened vulnerability of TGD adolescents to both disordered eating and suicidal behaviors compared to their cisgender peers. Among the TGD adolescent groups in this study, nearly 35% or more had experienced disordered eating in the last 30 days, with all gender minority groups having a significantly higher proportion than cismales and cisfemales. Though much of the research on TGD adolescents and disordered eating does not distinctly examine gender-specific groups (e.g., transfemale, transmale, nonbinary, etc.), the findings in this study appear consistent with other findings of significantly elevated rates of disordered eating in gender minority populations [1,4,22]. Based on the single item used to measure disordered eating, the eating behaviors in this study comprised both restrictive/compensatory types, which may offer new information about the higher prevalences of these types of behaviors than evidence has previously demonstrated [4].

The significant association between disordered eating and suicide suggests that these behaviors may exacerbate or be a symptom of exacerbated psychological distress. This finding is consistent with the literature, which identifies disordered eating as a maladaptive coping strategy for managing gender dysphoria and gender-related stigma, discrimination, abuse, and internalized transphobia [6,17,18,19]. The minority stress model [17] is frequently cited in TGD population research as a framework for increased mental health risks related to experiences of rejection, victimization, feeling unsafe, and non-affirmation [1,2,18]. Additional related factors that likely act as compounding stressors for TGD adolescents include having a fragmented family and lacking support from friends [23], social isolation, gender- or weight-based bullying [18,24], inadequate access to healthcare, discrimination by healthcare providers, lack of insurance coverage and multidisciplinary approaches, and delays to diagnosis and treatment [14].

The etiology of disordered eating and suicidality in TGD individuals is complex and multifaceted. TGD individuals may engage in disordered eating to alter their bodies, and these changes can potentially exacerbate or alleviate their gender dysphoria and related identity conflicts [25,26]. The negative health outcomes of disordered eating (e.g., malnutrition, fatigue, etc.) may intensify feelings of body dissatisfaction or gender dysphoria, creating an exaggerated sense of body dissonance [26]. Additionally, the stress and anxiety associated with maintaining disordered eating can heighten internal conflicts and contribute to a loss of control over one’s body and identity.

On the other hand, some TGD individuals may use disordered eating with the intention of achieving alignment between their body shape and gender identity [5]. For example, weight loss might reduce the prominence of secondary sex characteristics, such as breast tissue in transmasculine individuals. This alignment may provide a sense of congruence, potentially improving mental well-being. Though this concept seems logical, it suggests that gender affirmation and/or identity–body congruence might decrease disordered eating. Interestingly, preliminary evidence has not seen a significantly decreased prevalence in disordered eating among TGD adolescents over the first 12 months of accessing gender-affirming care [27]. More complex relationships may exist between gender identity and disordered eating, involving reward/punishment cycles [11,12,13]. As such, gender-affirming care clinical teams should consider the relevance of eating behaviors as potential treatment outcomes in addressing gender dysphoria. The complex relationships between eating behaviors, gender, and mental health emphasize the importance of policy-protected and accessible gender-affirming care for TGD adolescents with gender dysphoria.

Due to the limited research on this topic and the substantial health implications for developmentally sensitive adolescents, future research should aim to understand the underlying mechanisms contributing to the relationships between gender, disordered eating, and suicide, as well as how they may be related to one another. Specifically, more longitudinal research is needed to map eating behavior functions and trajectories for TGD adolescents who do and do not have access to gender-affirming care. Additionally, disordered eating among TGD adolescents should be conceptualized and investigated beyond the scope of simultaneous gender dysphoria, as it is not a universal experience in this population and likely not the only risk factor for disordered eating. For example, even in circumstances of accessible and effective gender-affirming care and/or treatment of gender dysphoria, other underlying factors contributing to disordered eating may include experiences of trauma, discrimination, victimization, discriminatory policy, etc. Likewise, decreases in disordered eating cannot be considered indicative of effective gender-affirming care.

Finally, with over 5% of the high school students in Colorado reporting themselves as identifying as a gender outside of the cisgender binary categories, more population-based studies need to prioritize best practices for gender measurement to adequately assess the needs of gender minority adolescents. Allowing students to accurately and safely identify themselves is a prerequisite to effective adolescent health promotion and intervention.

While our findings offer insight into the complex interplay between gender identity, disordered eating behaviors, and suicide risk among high school students, this study also has several limitations. The cross-sectional design of this study limits the ability for causal inference. Additionally, the HKCS collects self-reported data in alignment with the national YRBS [21] measures in school settings, which may involve a social desirability bias influencing responses. The disordered eating item only assessed restriction/compensatory behaviors. As such, the assessment’s questions for disordered eating and suicide are brief and not inclusive of clinically valid symptom scales. Additionally, the single item condensed several behaviors into one question (e.g., taking any diet pills, powders, or liquids; skipping meals, etc.), and thus the proportion of those who endorsed disordered eating may be inaccurately inflated. Further, the gender category of gender-questioning was used as the reference group in models 3 and 4 because the sample was the largest among the gender diverse groups (*n* = 1182); however, this may not be the most appropriate reference category. Finally, the race/ethnicity categories were condensed into one instead of examining each as distinct characteristics of the participants.

## 5. Conclusions

Population-based studies, such as the HKCS and YRBS, can offer critical data to support meaningful local, state, and national public health interventions. Importantly, the 2023 HKCS offers a unique ability to assess a state-wide sample of TGD adolescents given the inclusion of a two-step approach to gender measurement. Our study emphasizes the prevalence of disordered eating and suicide disparities observed among TGD adolescents. In particular, gender and disordered eating both significantly contribute to suicide risk among adolescents, with pronounced effects for gender minority subgroups. As such, researchers should continue to investigate the mechanisms that underlie these relationships, especially among heterogenous gender minority groups of adolescents, to better inform prevention strategies. Additionally, future research should investigate disordered eating behaviors with more specificity to particular behaviors (e.g., binge eating, etc.) and subsequent relationships with gender and suicidality.

This study underscores the importance of gender-affirming policies and practices in health systems and schools as a point-of-entry for risk identification, preventive services, and discerning acute needs. Eating behavior screening should be readily integrated into gender-affirming care and adolescent mental health services. With the high prevalence of suicidality among TGD adolescents, it is essential that researchers engage in innovative investigations of modifiable factors that influence the relationship between gender identity, mental health outcomes, and suicide. An overwhelming majority of TGD adolescents in the US have felt negatively impacted by recent politics, and nearly half of these adolescents and their families have considered moving to different states because of state-level legislation that endangers their access to healthcare. For the health and safety of TGD young people and their families, continued knowledge generation around TGD mental health is urgent and critical given the global and US political attacks that threaten or prohibit access to evidence-based medical care.

## Figures and Tables

**Table 1:** Demographic characteristics for the sample by suicidal ideation and attempt.

	Total Sample *n* (%)	Suicidal Ideation % (95% CI)	*p ^†^*	Suicide Attempt % (95% CI)	*p ^†^*
*N*	49,989	11.1 (10.2–12.2)		5.5 (4.9–6.1)	
Gender			<0.001		<0.001
Cismale	23,126 (48.3)	7.8 (7.4–8.1)		3.4 (3.1–3.6)	
Cisfemale	22,024 (46.0)	12.4 (12.0–12.9)		6.0 (5.7–6.4)	
Transfemale	189 (0.4)	36.3 (29.0–43.6)		21.4 (15.2–27.6)	
Transmale	502 (1.0)	44.1 (39.6–48.7)		19.8 (16.2–23.5)	
Nonbinary	886 (1.8)	37.4 (34.1–40.7)		16.7 (14.2–19.3)	
Questioning	1182 (2.5)	34.1 (31.2–47.0)		18.1 (15.8–20.5)	
Grade			0.390		<0.001
9th	14,758 (29.8)	11.0 (10.5–11.6)		6.2 (5.8–6.6)	
10th	13,534 (27.3)	12.0 (11.4–12.6)		5.9 (5.5–6.3)	
11th	11,677 (23.6)	12.3 (11.6–12.9)		5.1 (4.7–5.6)	
12th	9570 (19.3)	11.3 (10.7–12.0)		4.5 (4.1–5.0)	
Race			<0.001		0.001
White (non-Hispanic)	25,150 (52.2)	11.6 (11.2–12.0)		4.5 (4.3–4.8)	
Black (non-Hispanic)	1621 (3.4)	11.4 (9.7–13.0)		6.7 (5.4–8.0)	
Hispanic	9528 (19.8)	9.0 (8.4–9.6)		5.5 (5.0–5.9)	
Other	2775 (5.8)	11.6 (10.4–13.0)		7.2 (6.2–8.2)	
Multi-racial	9086 (18.9)	14.8 (14.0–15.8)		7.4 (6.7–8.0)	
Mother’s education			0.242		<0.001
High school or less	12,258 (30.2)	13.1 (12.5–13.8)		7.2 (6.8–7.7)	
Some College	5064 (12.5)	13.1 (12.2–14.1)		6.0 (5.3–6.7)	
College or graduate	23,226 (57.3)	10.9 (10.5–11.4)		4.4 (4.2–4.7)	
Disordered eating behavior			<0.001		<0.001
Yes	8953 (21.9)	28.6 (27.6–29.5)		15.3 (14.5–16.0)	
No	32,011 (78.1)	7.2 (6.9–7.5)		2.8 (2.6–3.0)	

Note: CI: confidence interval. ***^†^*** Pearson’s chi-squared test.

**Table 2:** Multivariable logistic regression modeling of gender and suicide outcomes and test of moderation by disordered eating behaviors.

	Total Sample (*N* = 44,540)		TGD Sample (*N* = 2486)	
	Model 1 OR (95%CI)	Model 2 OR (95%CI)	Model 3 OR (95%CI)	Model 4 OR (95%CI)
**Associations between Gender and Suicidal Ideation**				
**Main Effects**				
**Gender**				
Cismale	Ref.	Ref.	-	-
Cisfemale	**1.648 (1.537–1.767)**	1.005 (0.909–1.111)	-	-
Transfemale	**6.633 (4.673–9.414)**	**6.577 (4.153–10.418)**	1.054 (0.724–1.536)	1.250 (0.759–2.058)
Transmale	**9.611 (7.802–11.840)**	**7.288 (5.423–9.795)**	**1.495 (1.167–1.915)**	1.370 (0.963–1.950)
Nonbinary	**7.462 (6.331–8.796)**	**5.157 (4.049–6.567)**	1.181 (0.955–1.460)	0.980 (0.720–1.334)
Questioning	**6.408 (5.499–7.466)**	**5.311 (4.293–6.568)**	Ref.	Ref.
**Disordered eating behaviors**				
Yes	-	**3.557 (3.122–4.051)**	-	**3.122 (2.272–4.292)**
No	-	Ref.	-	Ref.
**Interaction**				
Cismale × DEB	-	Ref.	-	-
Cisfemale × DEB	-	**1.581 (1.344–1.859)**	-	-
Transfemale × DEB	-	0.540 (0.256–1.131)	-	0.622 (0.281–1.379)
Transmale × DEB	-	0.992 (0.628–1.567)	-	1.329 (0.646–1.909)
Nonbinary × DEB	-	1.030 (0.716–1.481)	-	1.164 (0.730–1.854)
Questioning × DEB	-	0.898 (0.639–1.264)	-	Ref.
Intercept	0.096 (0.084–0.110)	0.069 (0.059–0.081)	0.869 (0.587–1.287)	0.528 (0.336–0.832)
**Associations between Gender and Suicidal Attempt**				
**Main Effects**				
**Gender**				
Cismale	Ref.	Ref.	-	-
Cisfemale	**1.892 (1.705–2.099)**	1.126 (0.956–1.325)	-	-
Transfemale	**8.591 (5.672–13.011)**	**7.623 (4.114–14.126)**	1.436 (0.916–2.251)	1.700 (0.858–3.367)
Transmale	**7.140 (5.441–9.369)**	**5.649 (3.594–8.879)**	1.178 (0.856–1.620)	1.245 (0.726–2.137)
Nonbinary	**6.378 (5.114–7.953)**	**3.779 (2.531–5.642)**	1.063 (0.807–1.400)	0.837 (0.510–1.374)
Questioning	**6.098 (4.987–7.456)**	**4.544 (3.271–6.312)**	Ref.	Ref.
**Disordered eating behaviors**				
Yes	-	**4.346 (3.594–5.256)**	-	**3.651 (2.411–5.528)**
No	-	Ref.	-	Ref.
**Interaction**				
Cismale × DEB	-	Ref.	-	-
Cisfemale × DEB	-	**1.512 (1.193–1.916)**	-	-
Transfemale × DEB	-	0.644 (0.269–1.543)	-	0.768 (0.297–1.987)
Transmale × DEB	-	0.760 (0.421–1.374)	-	0.892 (0.443–1.797)
Nonbinary × DEB	-	1.063 (0.638–1.769)	-	1.265 (0.673–2.378)
Questioning × DEB	-	0.854 (0.544–1.341)	-	Ref.
Intercept	0.056 (0.046–0.068)	0.036 (0.029–0.045)	0.368 (0.222–0.608)	0.198 (0.108–0.361)

Note: Model 1: multivariable analysis with gender and covariates, including sociodemographic characteristics (grade, race, and mother’s education). Model 2: multivariable analysis with gender and covariates in model 1 along with the interaction term gender × disordered eating behaviors. Model 3: multivariable analysis with gender and covariates including sociodemographic characteristics (grade, race, and mother’s education). Model 4: multivariable analysis with gender and covariates in model 3 along with the interaction term gender × disordered eating behaviors. Bold: *p* < 0.001. TGD: transgender and gender diverse. OR: odds ratio. CI: confidence interval. DEBs: disordered eating behaviors.

## Data Availability

The original data presented in the study are openly available at the Colorado Department of Public Health and Environment at https://cdphe.colorado.gov/healthy-kids-colorado-survey-dashboard accessed on 22 April 2024.
